# Time-resolved ultra-weak photon emission as germination performance indicator in single seedlings

**DOI:** 10.1016/j.jpap.2020.100001

**Published:** 2020-03

**Authors:** Cristiano de Mello Gallep, Daniel Robert

**Affiliations:** a School of Technology, University of Campinas, r. Paschoal Marmo 1888 – 13484-332, Limeira – SP / Brazil; b Life Sciences, University of Bristol, 24 Tyndall *Av*. - BS8 1TQ Bristol / UK

**Keywords:** Germination vigour, Low-level luminescence detection, Spontaneous light emission

## Abstract

As they grow, plant seedlings emit very small amount of light, the so-called ultraweak photon emissions (UPE). Numbering tens or hundreds of photons per second, this UPE radiation has been measured on groups of 10 to 1000s of seedlings growing together. Here, we set out to measure UPE on single germinating seeds, enabling the assessment of individual seeds. Here, UPE were measured in low-noise laboratories, enabling long-term, stable and reproduceable testing conditions. UPE recordings could in effect be performed on single germinating seedlings of mung beans, corn and wheat, also comparing measurements to 10-seed sample of mung beans. The data reveal differences between seedlings and plant species. The relation between the seedling's development – total length of roots plus leaflet - and the UPE data is presented in terms of the total photon counts as a function of seedling growth in time. These data were fitted in linear interpolation, for the tests with mung beans when related to the UPE slope (R^2^ ~0.834). UPE *versus* growth correlation was weaker for tests conducted with single mung seedlings (R^2^ <0.4), revealing individual variation and seedling movements during growth. For single corn, intermediates correlation coefficients R^2^ were observed (0.57<R^2^ <0.83), while single wheat seed data were more sparse (R^2^~0.62). Altogether the data show a general pattern of linear UPE to growth relationship, yet highlighting variations between plant species, which provide a benchmark for assessing the health of early seedlings.

## Highlights

• It is possible to measure the UPE of single seedlings of wheat, corn and mung bean.
• Repeated measurements show direct relation between UPE and seedling growth.

## Graphical abstract

**Figure:**
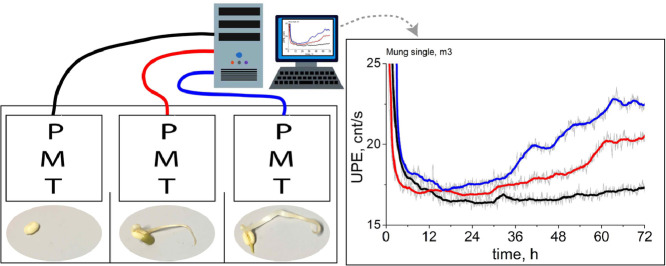

## 1 Introduction

As early 1920, A. G. Gurwitsch proposed that light emission would occur during seed development on the basis of chemical reactions in plants [1]. Lacking empirical evidence, this claim was only experimentally verified with the subsequent advent of sensitive photon counting (PC) devices in the 1950s, such as the photo-multiplier tube (PMT). Colli and Facchini [2] were the first to demonstrate that groups of seedlings produce photon emission during growth. Located in the human visible light spectrum, this UPE increases with time, whereby the young roots were identified as the main source of the radiated energy [2,3].

Since then, more recent measurements have reported ultra-weak photon emissions (UPE) across many taxa, and it has been proposed that UPE occur in all living organisms, spanning a spectrum near UVA, visible light and near IR [4,5]. Lacking a parsimonious terminology, UPE has received several denominations, such as weak luminescence, endogenous luminescence, spontaneous chemiluminescence, biophoton, amongst other terms [4].

Phenomenologically, UPE has been regarded as a by-product of the metabolic production of reactive chemical species, mainly radical-oxygen species (ROS) that are electronically excited and that occur inside living organisms and tissues. These photon emissions are related to the production of triplet excited carbonyls and singlet oxygen (^1^O_2_) species released during enzymatic reactions, such as catalasesm, oxygenases and peroxidases [4,6]. As detailed in ref [4]., the current hypothesis is that ROS react with biomolecules and give unstable intermediates as result - such as dioxetane and tetroxide - that when decomposing would form triplet excited carbonyls and singlet oxygen, that can emit photons in the range of 350–550 nm and of 550–750 nm, respectively. In effect, UPE occurs during both normal development [7] or in response to alterations of the organism's normal physiological state [8]. So, while the organism grows or reacts to environmental inputs, it glows. UPE have been recorded when a plant's immune system engages in defence, or tissue responds to physical perturbation or chemical injury [4,^8^,9]. In the case of intact seedlings, it was show that the main emitters are the rootlet's base and tip, regions where the metabolism is more active due to cell division and elongation [7].

Also related to this phenomenon is the ultra-weak delayed luminescence (DL). DL occurs after exposure to a brief light pulse and has been applied to quickly assess viability of groups of seeds [10,11] or to the rapid and regular assessment of water toxicity after inoculation with toxic algae [12].

Long term, day-length recordings of spontaneous UPE during the development of seedlings were first presented by Yan in 2006 [13]. Shortly after, UPEs were shown to be analytically useful by the Laboratory of Applied Photonics in Campinas, Brazil [14,15], as light emissions constitute reliable indicators of seed germination and development in chronic toxicological testing conditions, with more developed seedlings presenting more photon counts. Under acute stress, when a chemical stressor is applied after the first days of germination, the response is the opposite, with the more damaged, less developed sprouts presenting higher photon-counts, just after the stress, than the control samples, and the burst lasting for few hours (6–12 h) [16]. Our group also presented evidence that non-stressed samples presented daily and monthly oscillations behaving similar to the local gravimetric tide profile, with coincident pattern inflections and similar periodic components for different species [17,18].

The results above stem from germination UPE using seed samples ranging from 6 to *ca*. 50 seeds of wheat, bean or corn in each trial. Through such collective measurements, the pooling of UPEs were only then sufficient to exceed the detector (PMT) darkcount noise and produce reliable signals. Recently, using a large collective sample of *A. thaliana* seeds (~7000), long term UPE recording of *Arabidopsis thaliana* was presented for the first time, reporting the characteristic enhancement of UPE following acute chemical injury [19].

In order to further understand the mechanisms of UPEs, time-resolved dynamics and vulnerability, we set out to measure series of long term UPE records of single seedlings, across early developmental stages for mung bean, corn and wheat. We also present results on ageing mung bean seeds, yet measured on 10-seed samples. For all species, we also investigate the relation between seed development, measured as total root and leaflet linear length, and UPE time data. A linear relationship was used to quantify the fit between the observed sample growth and its UPE, revealing a good correlation for the 10-seed samples (R^2^ ~0.834), but a weaker linear correlation for single mung seedlings (R^2^ <0.4). In effect, the movements and positional changes of mung's sprouts as they grow is deemed responsible for the increase in UPE variation and reduction in correlation. The series of single corn exhibited intermediate linear correlation (0.57<R^2^ <0.83), while single wheat time series revealed loser correlations (R^2^ ~0.62).

## 2 Material and methods

Germination tests were performed inside a dark box (H:30 cm L: 30 cm W: 34 cm) standing in a semi-dark room (H:2.4 m L: 3.45 m W:3.20 m), ensuring a minimal background photon count, on the order of the PMTs’ dark-count noise (<15 counts/s). Hence, experimental conditions allowed a sensitive and reliable characterisation of base line photon counts. The experimental room was located in a deep basement floor, with its walls made of concrete lined with earthed double layer aluminium acoustic panelling, thus minimizing spurious effects of electromagnetic noise and cosmic rays on the sensitivity and reliability of the PMTs. Temperature was controlled to be 21±2 °C throughout all experiments. Experimental protocols ensured that light ingress during long time series was guaranteed not to happen. All experiments were conducted on a vibration isolation optical table (Thorlabs PFA51501 model, 1.5mX0.75 m), itself positioned on a vibration-isolation inertial 8-tons concrete block to minimise external mechanical inputs to the seed and PMT measurement setup.

### 2.1 Germination series

Germination tests were performed for mung beans (*Vigna radiata*), corn (*Zea mays*) and wheat (*Triticum aestivum*), using sterile polystyrene petri-dishes (35 mm x 10 mm, 430,165 Corning Inc.). Filtered water was used to initiate germination. Seed stocks were kept in dark and prepared under minimal light to avoid long delayed luminescence at the beginning of PC measurements. Each dish with one imbibed seed (or a group thereof) was put under each of the three PMT sensors to perform photon-count measurements during sprout development. At the end of the measurement period, seedlings were photographed and analysed by measuring total length (leaf and/or root) of samples and these data related to their corresponding individual UPE time series. Germination tests were performed with 3 simultaneous sample seed each time, using 3 sensors in parallel - ch0, ch1 and ch2 -, eg. one for each seed germination test. Seeds were chosen randomly from visually intact ones; details of stock and supplier are presented at Appendices A to D.

Mung beans were tested initially using collective samples of 10 seeds each for the *‘S‘ series* - S1 to S6 - using intact control samples ‘t0’ as well as samples that suffered thermal stress – ‘t1’ and ‘t2’, testing for differences in germination vigour. The thermal treatments were performed in controlled dry incubators to promote seed ageing; parts from the same main stock used as control (‘t0’) were kept at 100 °C for 30 min for treatment t1, and for 50 min for the treatment t2.

For this *S-series*, rounds S1 to S4, a sample of 10 t0 seeds was germinated under the ch0 PMT sensor, a sample of t1 seeds under ch1 sensor and a sample of t2 under ch2; for round S5 all three samples were taken from t0 stock, and for S6 all were taken from t1 stock. All S-series tests used 3 mL of filtered water. A second series of mung bean germination – the *m-series*, m1 to m6 – were performed using a single seed under each of the three PMT sensors, all three seeds of each round taken from the intact control group (t0) and imbibed in 2 mL of water. Both mung series had PC measurement starting from the moment just after their imbibition and running continuously for the next 72 h.

Corn germination series used single seed samples of pop corn (*c-series*, c_1 to c_7, 1.5 mL of water). No stress was applied to the seeds’ stocks. The germination tests did last for 6 days; the PC measurements started 72 h after imbibition for all rounds. All samples were stored covered in aluminium foil just after the imbibition and so kept in darkness until the move to the dark chamber. Only then, the foil was removed under minimal light, as to avoid sample illumination and long term DL.

A wheat germination series – w1 to w6 – used single, intact seeds and 1.5 mL of water in each test, with PC measurement starting just after imbibition and lasting for 72 hrs.

### 2.2 Photon-count setup

The UPE of the germination tests were measured using photon-count units (PCU) - model H11870–01, Hamamatsu Photonics KK – that integrates high-voltage power supply and photon-multiplier tube (PMT) sensitive to the visible range (300–650 nm, selected units for low-dark noise (<15 counts/s). No optical filtering was applied, so photon-count data refers to the entire visible and near UV spectra where the PMT's photocathode is responsive. The three PCU (channels ch0, ch1 and ch2) were equivalent in their basal dark count and sensitivity, and were used simultaneously during the 3 simultaneous tests of each time series. The dark-count noise levels of the three PCU devices were measured continuously for 24 hrs and the average value used to calibrate PCU's signal. In order for photon count data to be numerically comparable between the germination tests, calibration factors of 1.07 and 0.92 were used for ch1 and ch2 signals respectively, in effect normalising basal levels to that of ch0. Altogether, the total darkcount for a 24-h period is around 1.2 × 10^6^ counts.

Photon count data were acquired using a dedicated software controlling a counting-board (C8855–01, Hamamatsu KK) for each of the three PCU channels. Counts were integrated for a 1 sec time window and data were streamed directly and stored on hard disk. A typical time series of 3 days was usually made of 259,200 data points.

Similarly to the setups used in the past [14,15], PCU devices were positioned vertically with the PMT's photocathodes just over the petri-dish, but here a very small gap (~1 mm) between the frontal sensor window and the dish lid was used. This configuration was implemented as to maximize the number of photons captured the photocathode, that is so entirely exposed to the area where the seedling develops, inside the dish. So, photons originated in all parts of the seedling - initially the germ, and latter rootlets and leaflets - might contribute to the total photon-count data. Accurate sample positioning also enable sufficient ‘photon harvesting’ for the time-resolved UPE measurement of single developing seedlings.

### 2.3 Data analysis

At the end of each series’ round, the PC data (cnt/s) were plotted for ch0, ch1 and ch2, using the local smoothing average of 1000 points to discard thermal noise fluctuations inherent to such measurements. PC data of each test were also condensed to a pair of values in terms of the:

- Sum UPE_,_ sum of photon-counts for the entire period of each test, or for a partial period such as the last day of each test, when counts are higher;
- UPE slope, linear growth in time, ie. the slope of the best linear fitting of each PC curve.

To perform such linear fit, the initial DL in the UPE profile, if present, was discarded by leaving the first 2 hrs of recording out of the analysis. This procedure was sometimes necessary for mung beans and wheat series that, because PC acquisition started just after imbibition, implying the presence of DL. For corn, because seeds were stored and germinated in darkness, data presented only occasional, and if so much weaker DL and therefore did not require truncating initial recordings.

PC data – UPE sums and slope - were used in datagrams plotted *versus* the related total seedlings’ length – root for the mung series, and root + leaf for the corn and wheat series, and such datagrams interpolated by a linear fit for a first approximation. The UPE sum x total length linear fittings were also calculated for each round of 3 seedlings, and compared to the global fit, as presented in the correspondent Appendix.

## 3 Results and discussion

All UPE curves and seedlings pictures are presented in the Appendices, together with plots of UPE data *vs.* total length of seedlings.

The data gathered from all seed species indicate that the more vigorous germination is, the more substantial is the emission of ultraweak photons. This relation was first highlighted in measurements of wheat seeds measured in batches of 10 to 50 [15]. Previous tests inducing mild toxicity showed differential vigour has an effect on the time-dependant collective UPE. Here, the UPE of individual seedlings were recorded with high-resolution time series, under unaltered growth conditions. For all seed species tested, the UPE temporal organisation of individual seedlings is similar but not identical.

**Fig. 1:**
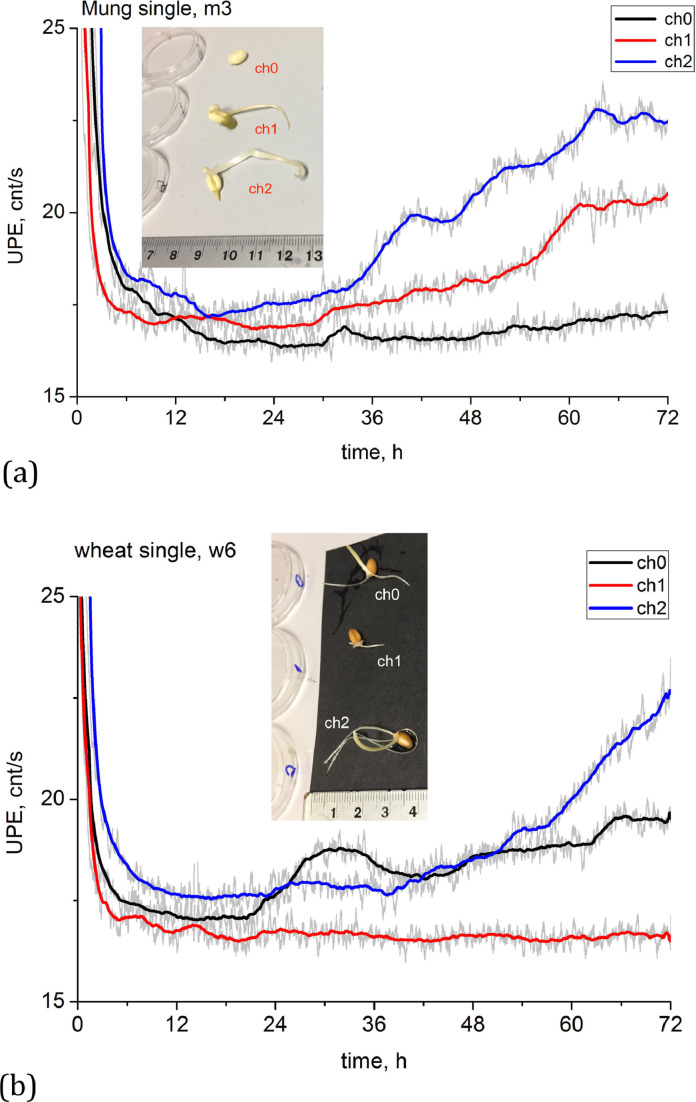
Photon emissions as a function of time, measured in count/s. (a) mung (test m3) and (b) wheat (test w6). Coloured curves, as roving averages of 10^3^ points, show different seedlings measured simultaneously by three independent channels, ch0, ch1, ch2. Insert shows pictures of each seedling at time of test termination (72 hrs).

First, for individual mung beans and wheat seeds, time-resolved data reveal that more developed seedlings present higher total counts of UPE but also a higher cumulative rate (Fig. 1, tests m3 (a) and w6 (b)).

By contrast, less well-developed seedlings present lower UPE total counts, as well as a flatter profile, a mark of reduced growth (ch0 for Fig. 1a; ch1 for Fig. 1b). Expectedly, seedlings with an intermediate growth present commensurate UPE total counts and time profiles (ch1 for Fig. 1a; ch0 for Fig. 1b).

**Fig. 2:**
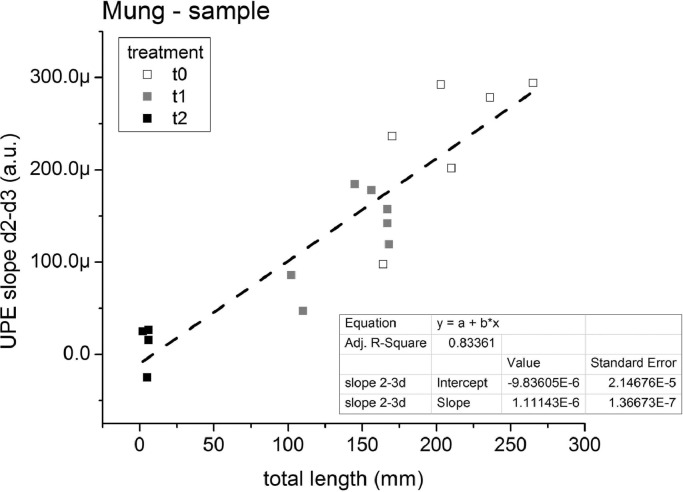
Mung sample germination tests (S1 to S6) showing linear UPE growth for the last two days (24h-72hrs: UPE slope d2-d3) *versus* total seedlings’ length (mm); Inset: linear correlation parameters.

This direct relation between UPE and sample development was confirmed for the S-series, for samples of 10 mung beans using control (t0 stock) and also artificially aged, dehydrated seeds (stocks t1 and t2). All UPE profiles are presented in Appendix A, as well as UPE *vs.* length datagrams; here Fig. 2 shows the plot of the UPE linear growth for the last two of the 3-day test (slope d2-d3) *versus* total seedlings’ length, where the well developed (t0), intermediate (t1) and non-developed (t2) samples are well clustered in this datagram, that presented the best linear approximation for this series: R^2^ = 0.83361. The datagrams using the total UPE (Sum_UPE) and the semi-final day UPE (Sum_48–60 hrs) presented more disperse points, with lower linear correlations, with respectively R^2^ = 0.54884 and R^2^ = 0.76295 (see Fig. A.1, Appendix A).

When measuring single seedlings, however, a direct linear relation between UPE growth can be harder to find in the global data, as exemplified by mung m-series: all global datagrams presented lower linear correlations for this series, with points well scattered and R^2^ < 0.34. The rate of UPE increase in all cases also exhibits significant scatter (Fig. B1, Appendix B). There is some variation in the UPE measurements, with some tests (eg. m3, Fig. 1a) presenting a different picture from tests m4 and m5, for which less developed seedlings presented higher UPE counts (Appendix B). However, when analysed in terms of each round of 3 seedlings (excluding round m4 that presented similar development for all sprouts), the relation holds: the bigger the final seedling the higher the photon count (Fig. B2, Appendix B), with the linear interpolations presenting similar slope for all runs.

**Fig. 3:**
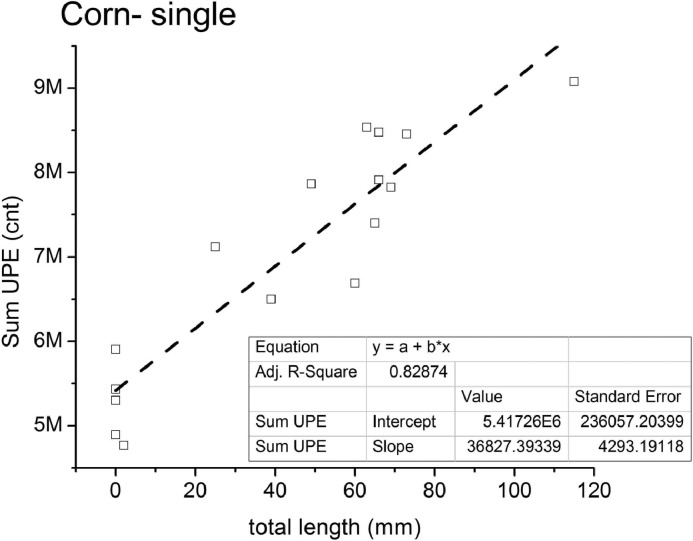
Single corn germination tests (c1 to c7) shown as datagram of the total photon-count (Sum UPE, cnt) for the last 3 days of germination *versus* the total seedlings’ length (leaf+root, mm); inset table: linear correlation parameters.

A good linear relation between UPE *vs.* growth was observed in the single-corn series (Fig. 3) whereby the sum of total UPE *versus* total seedling length for the c-series, yielded a correlation coefficient of R^2^ = 0.82874 for the global data. When related to the UPE sum at the last day (d6) and to the UPE slope (linear growth), it presented R^2^ = 0.82617 and R^2^ = 0.56669, respectively (Fig. C1, Appendix C). When analysed in terms of each round, the corn-series also presented very similar fitting slopes for the total UPE *x* seedling length, but round c3 (Fig. C2, Appendix C).

**Fig. 4:**
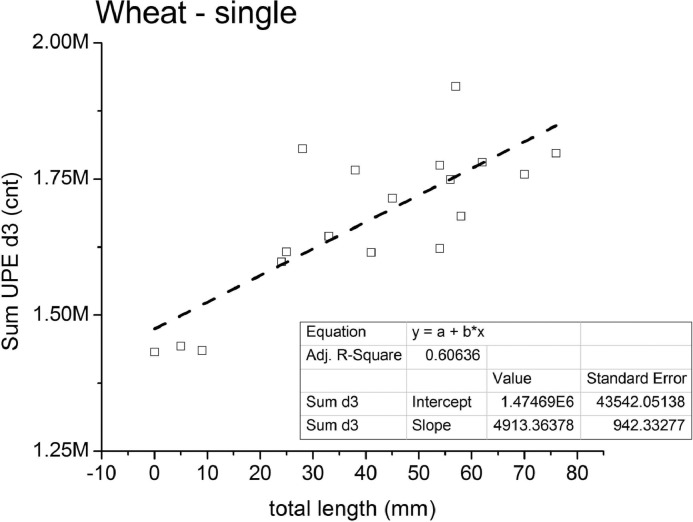
Single wheat germination tests (w1 to w6) - datagram for the total photon-count for the last 24 hrs period (Sum d3, cnt) *versus* the total seedlings’ length (mm); linear approximation with parameters in inset table.

The w-series for single wheat sprouts presents clear examples of direct relation of UPE *vs.* growth in the global data, as shown for test w6 (Fig. 1b). Yes, it is good to note that seedlings with similar development can present quite different UPE profiles (tests w4, w5 in Appendix D). The linear relation between seedling growth and UPE sum calculated for the last day (d3, ie. 48 hrs to 72 hrs) in the global data yield a loose correlation R^2^ = 0.60636 (Fig. 4) with the other two datagrams presenting total UPE and UPE slope, with R^2^ = 0.58292 and R^2^ = 0.48566 respectively (Fig. D.1, Appendix D). Also the analysis done in each round shows a variety of fitting slope, but nevertheless the more developed seedling did consistently present higher photon-counts (Fig. D.2, Appendix D).

Long term variations in UPE measured from multiple seedlings have been noted previously in tests [17,18]. For single seedlings, one possible cause of UPE variation could be the different position or orientation of the growing seedling relative to the PMT active cathode. It is possible that different morphological regions of seedlings contribute different UPEs. As seedlings twist and move around during growth, and sometimes even tumble, photon capture by the PMT unit may change, detecting only some portion of the UPE source [7]. The question thus arises as to whether and how heterogenous and directional UPE generated by seedlings are. Current PMT sensors cannot help resolve this geometrical optical problem. As seedlings emit more photons as they grow, it is possible that some of the UPE intensity is lost in different relative position, as some light emitting regions, even transiently, do not radiate in the direction of the PMT sensor. In tests multiple seed samples, this position uncertainty is reduced because the probability of several seedlings radiating photons in PMT direction is higher, constituting a more stable average source and presenting an apparent higher reproducibility [14,15].

## 4 Conclusions

The UPE long term time profiles of single seedlings, in series of germination tests for mung beans, corn and wheat are presented for the first time, together with a series of 10-seed samples for mung beans.

It was found that UPE data can be related to seedling development in a linear manner at first approximation (R^2^ > 0.7–0.8). Yet, for single seedlings, this linear relation can vary for the global data, pointing to possible geometrical aspects of UPE. Collectively, all tests conducted exhibit a direct relation between growth and UPE counts.

As UPE are related to metabolic activity, it is likely that only a small area on the seedling is responsible for light emission, perhaps a few µm^2^ or smaller. As root growth dominates the first developmental stages, it can be speculated that the root expansion zone, where cell division and growth are highest contributes most to UPE. Similarly, at a later stage the base of the leaflet is expected to produce photons thus adding a UPE generation site and changing geometry in relation to the PMT. Current PMT technology is such that lensing UPE to capture photons through optical imaging is not practical or sensitive enough. Establishing a spatially-resolved image of UPE in seedlings with small integration time (ie. better time resolution) and lower background noise than currently achieved [7,9] is the next step to further understand the structure and dynamics of photon emissions in growing plants. The future advent of single-photon imaging cameras would find another application in the spatially and spectrally-resolved measurements of UPE, and not only in seedlings. With improved single-photon imaging, the study of seed health, germinability, and response to environmental stress could be performed on single seedlings, enabling novel non-invasive measurements of metabolic activity at the tissue and cell level. Looking ahead, such UPE measurements could provide precious information about normal development processes, but also enable novel insights into plant responses to attacks by micro-organisms and environmental stressors.

## Contributors

CMG and DR have designed the experiments and written the text; CMG conducted experiments and data analysis.

## Data statement

The authors declare that the raw data is available under request.

## Declaration of Competing Interest

The authors declare that they have no known competing financial interests or personal relationships that could have appeared to influence the work reported in this paper.

## Appendix Supplementary materials

Image, application 1

Image, application 2

Image, application 3

Image, application 4
