# Supplementary material for: Time-resolved ultra-weak photon emission as germination performance indicator in single seedlings
Source: J Photochem Photobiol. 2020 Mar;1:100001. doi: 10.1016/j.jpap.2020.100001 (PMC7446287; doi:10.1016/j.jpap.2020.100001)
Supplement: Supplementary file 1 [file mmc1.pdf]

https://doi.org/10.1016/j.jpap.2020.100001

Table A.1 – Mung sample series S_i

Photon-count time profiles (local average, 1000#), total seedlings' length (error = +/- 1mm) and photograph at end of the 3-day germination tests of each trial of 3 samples (10 seeds each, 3 mL water): photon-count chamber ch0 with seeds of treatment t0, ch1 with t1 and ch2 with t2, unless for S5 (only t0 samples) and S6 (only t1 samples) (organic mung beans, *Essential* stock 01635-P165X). Technical failure during S4 lost some short periods, and so total count for this case was discarded.

Trial	Photon-count profiles	Total Seedlings' length	Picture
S1	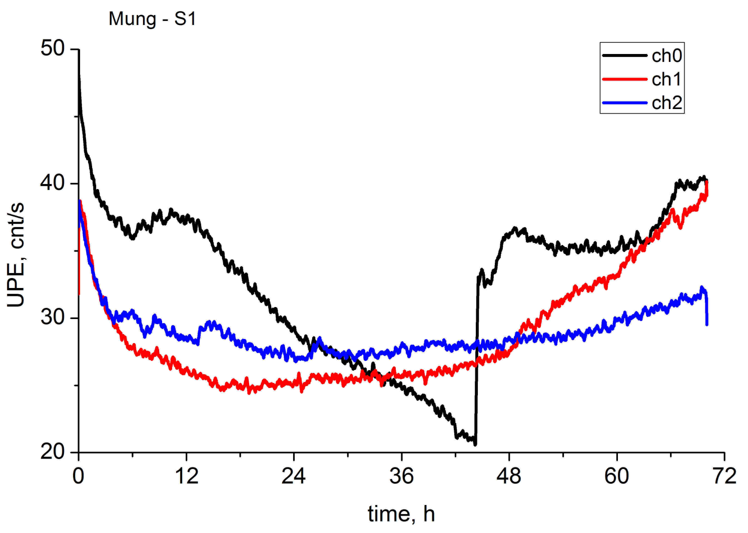	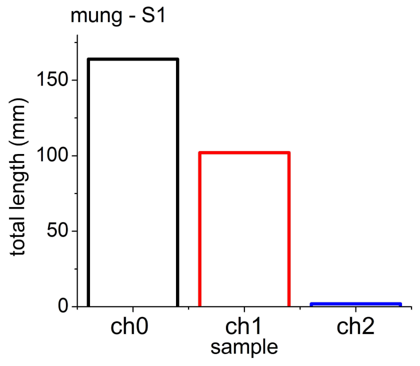	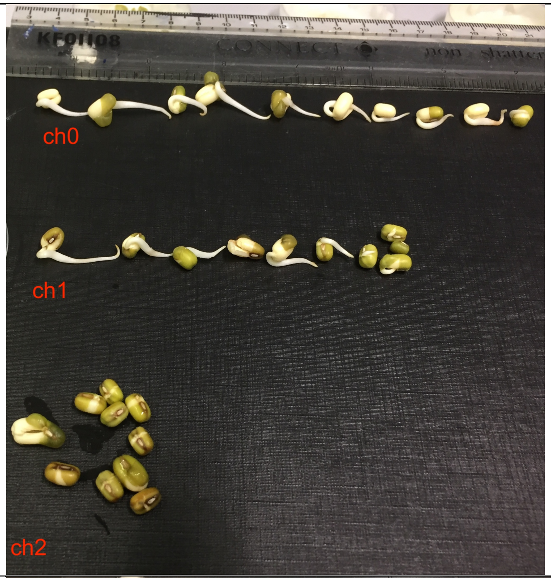
S2	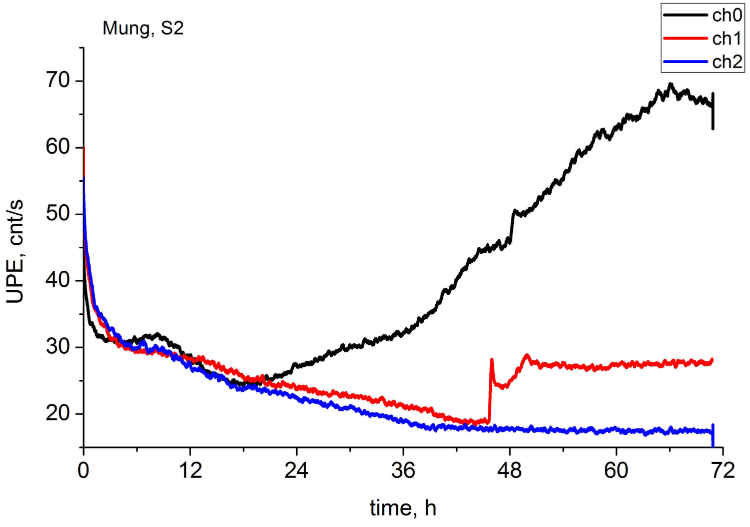	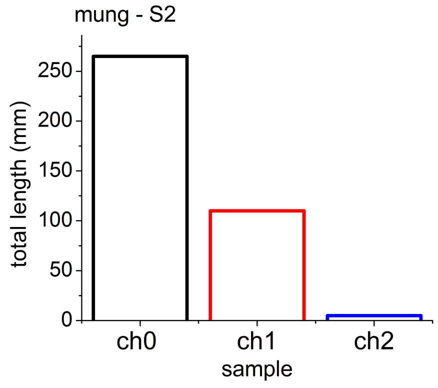	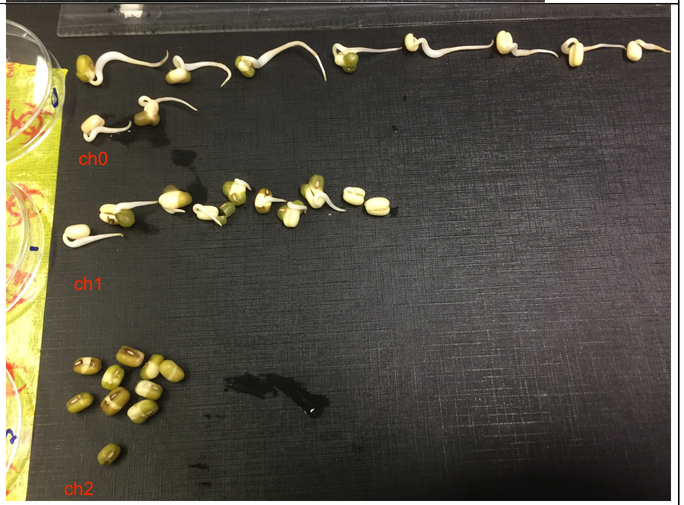

S3

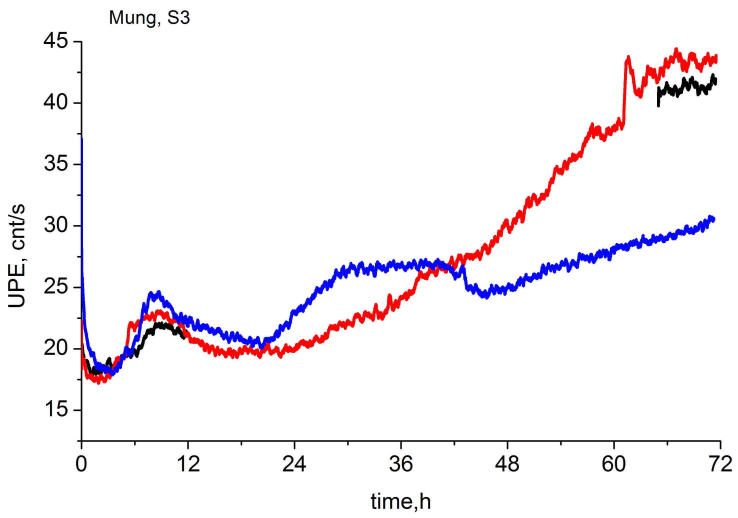

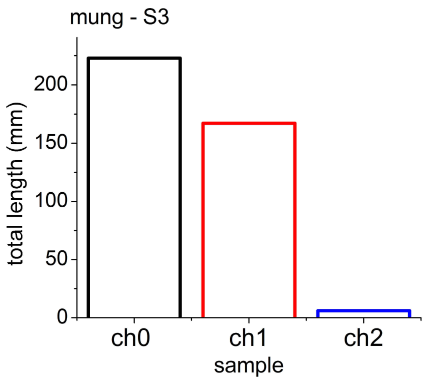

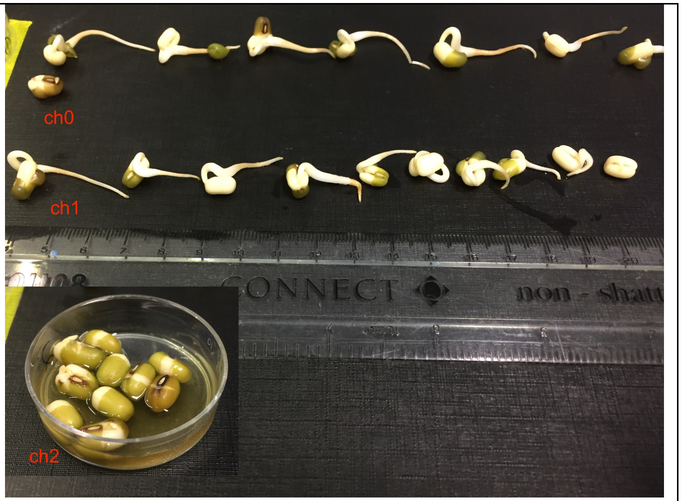

S4

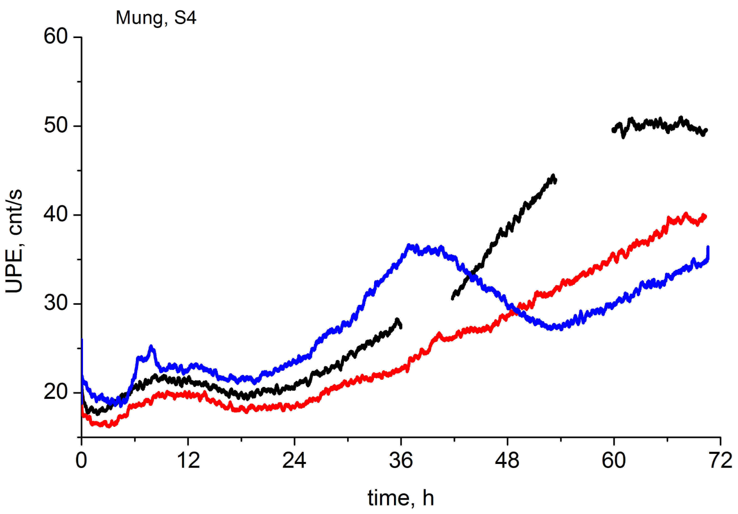

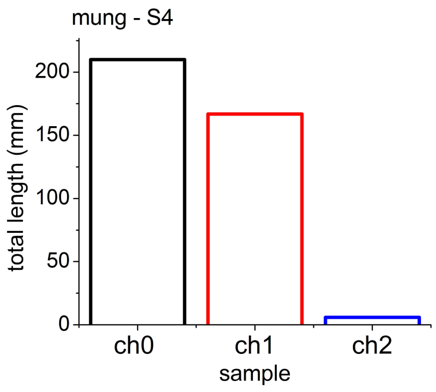

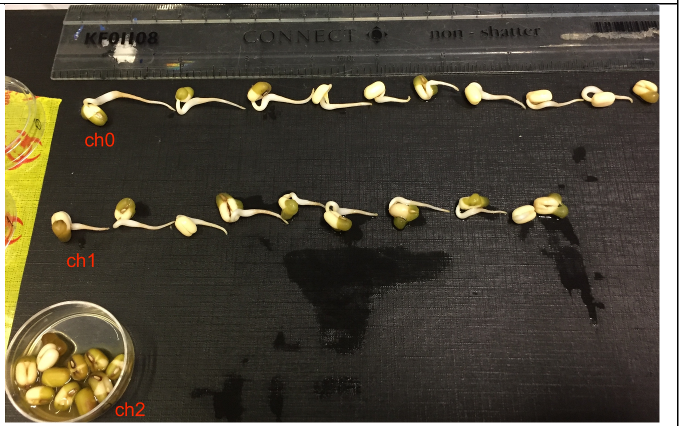

https://doi.org/10.1016/j.jpap.2020.100001

S5

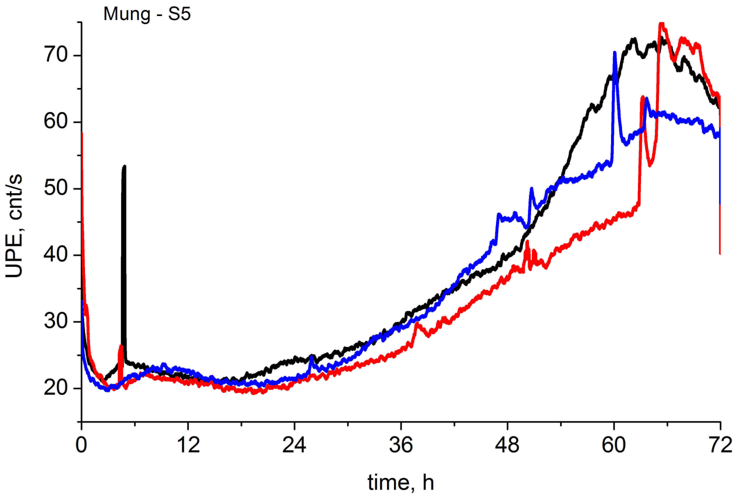

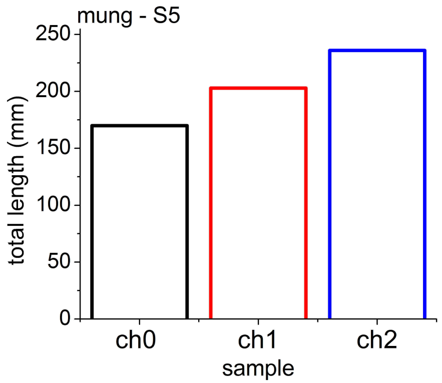

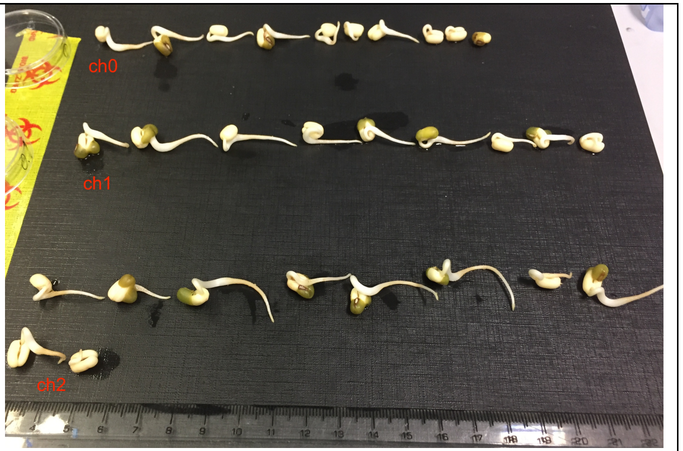

S6

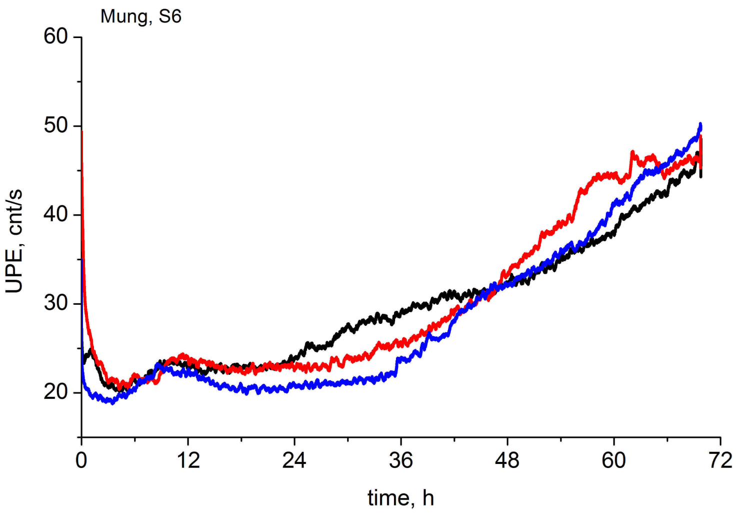

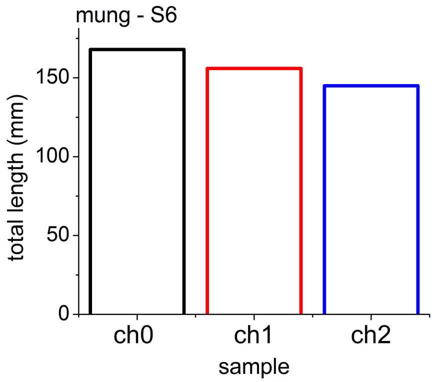

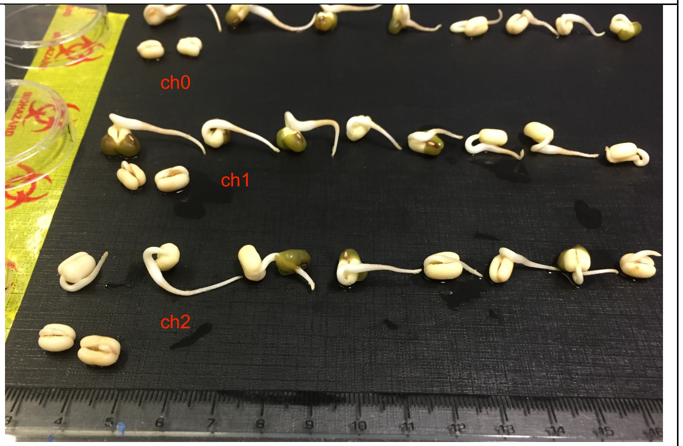

https://doi.org/10.1016/j.jpap.2020.100001

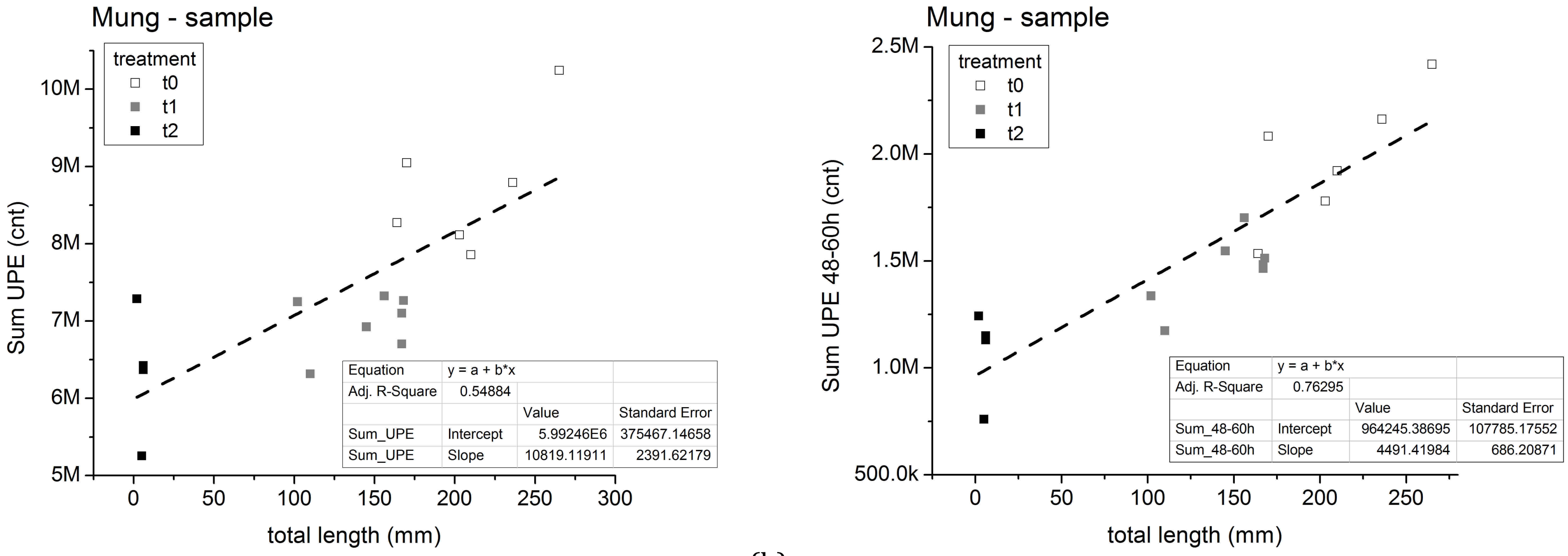

(a) (b)

Figure A.1 – Mung sample germination tests (S1 to S6) - datagrams of UPE data *versus* the total seedlings' length for the: (a) total photon-count (Sum UPE) for the entire period: 0-72h; (b) counts for 12h period (Sum UPE 48-60h); linear approximation with parameters at inset table.

https://doi.org/10.1016/j.jpap.2020.100001
