# Supplementary material for: Time-resolved ultra-weak photon emission as germination performance indicator in single seedlings
Source: J Photochem Photobiol. 2020 Mar;1:100001. doi: 10.1016/j.jpap.2020.100001 (PMC7446287; doi:10.1016/j.jpap.2020.100001)
Supplement: Supplementary file 2 [file mmc2.pdf]

Appendix B – Single Mung series

Table B.1 – Single mung series m_i

Photon-count time profiles (local average, 1000#) and photograph at end of the 3-day germination tests of each trial of 3 samples: photon-count chambers ch0, ch1 and ch2 with single mung seedling in petri-dish + 2mL of water (organic mung beans, Essential stock 01635-P165X).

<table>
<tr><th>Trial</th><th>Photon-count profiles</th><th>Picture</th></tr>
<tr><td>m1</td><td>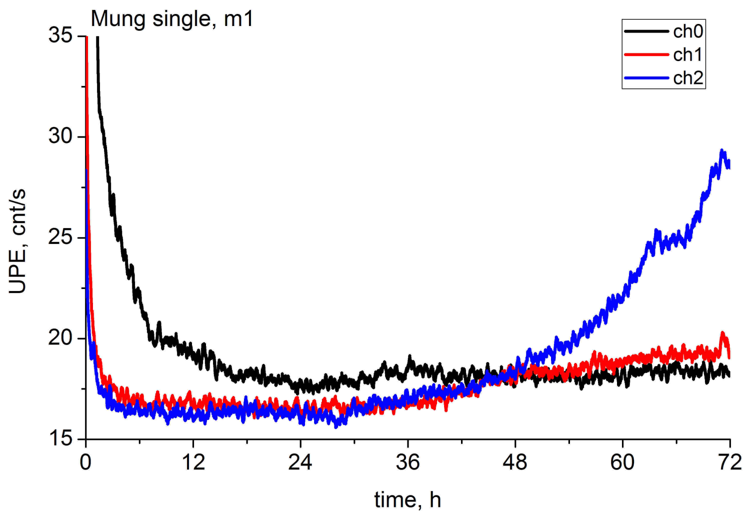
</td><td>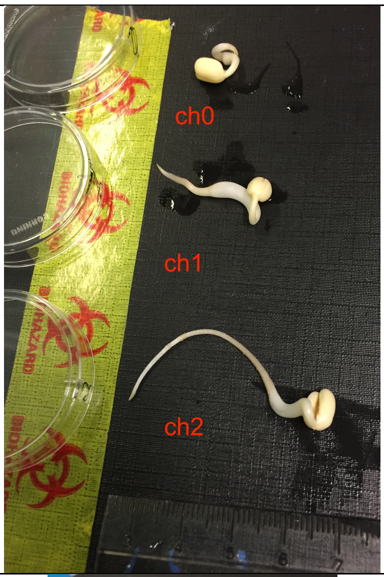
</td></tr>
<tr><td>m2</td><td>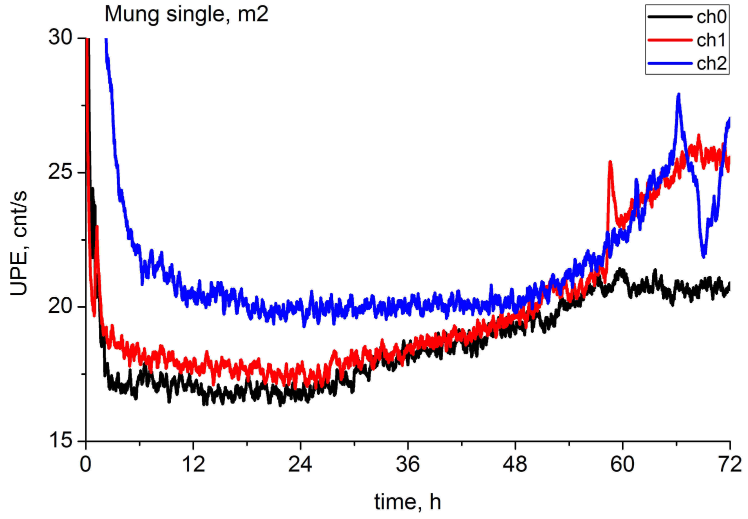
</td><td>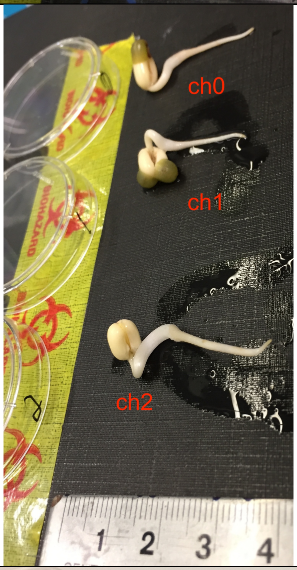
</td></tr>
<tr><td>m3</td><td>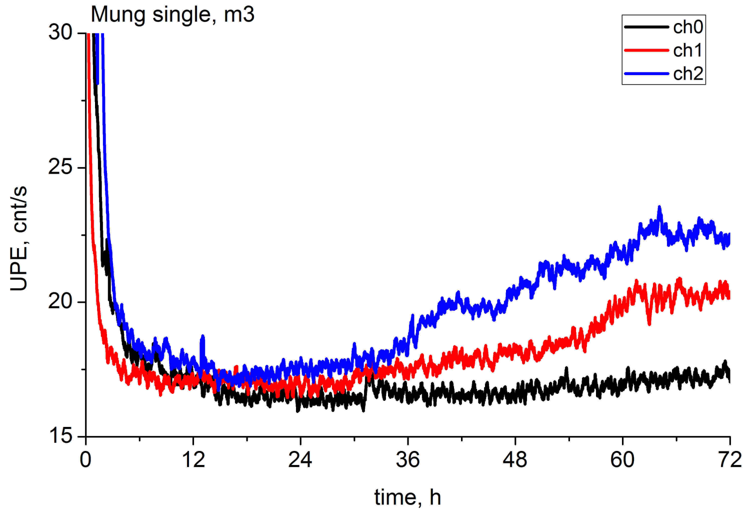
</td><td>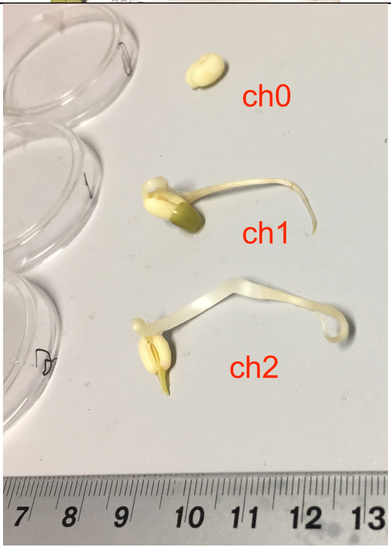
</td></tr>
</table>

https://doi.org/10.1016/j.jpap.2020.100001

m4

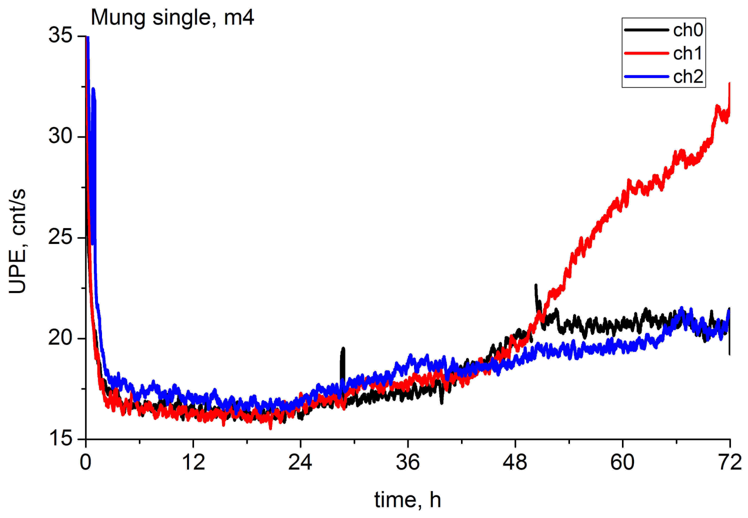

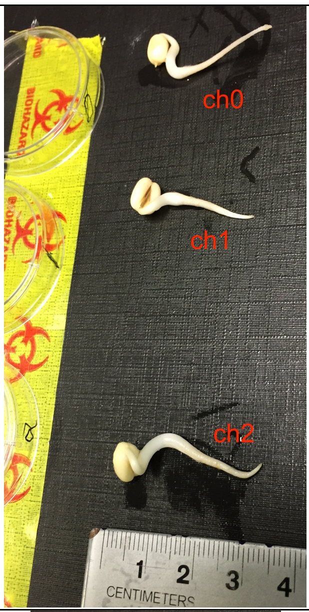

m5

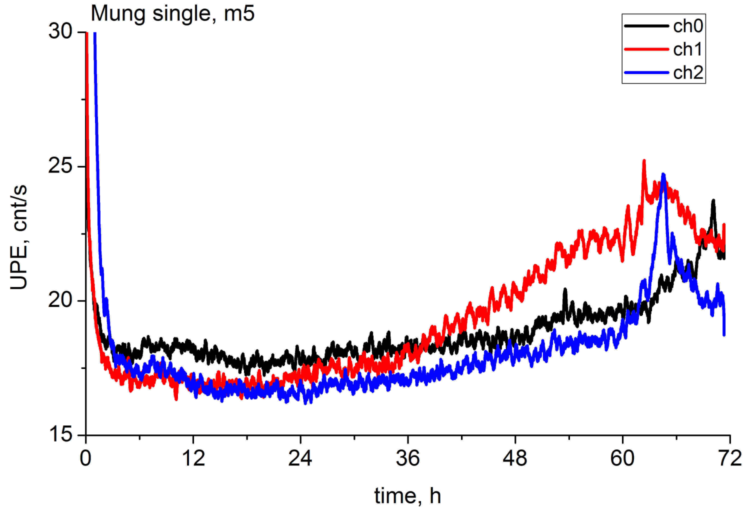

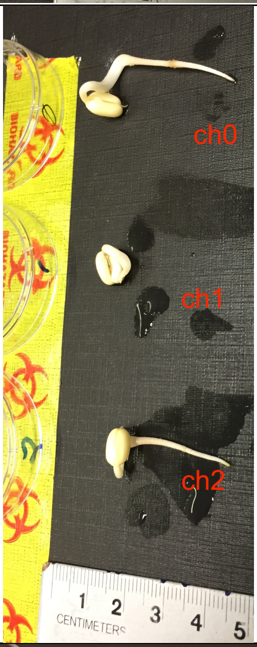

m6

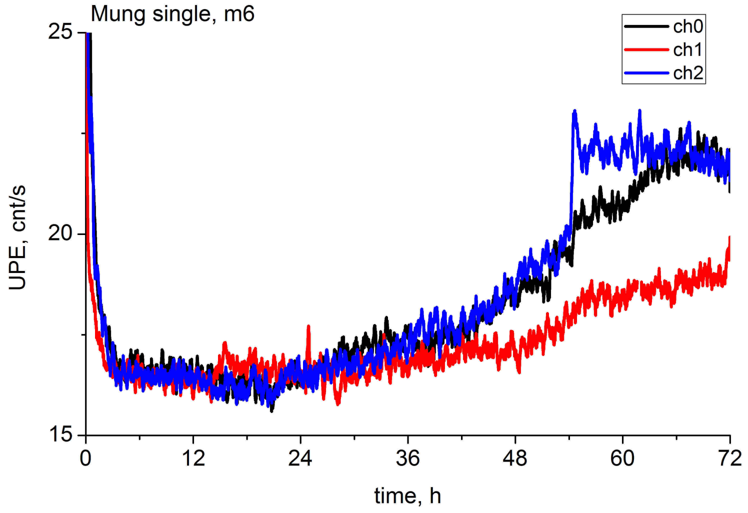

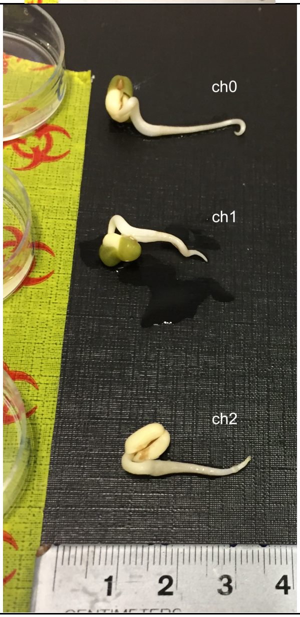

https://doi.org/10.1016/j.jpap.2020.100001

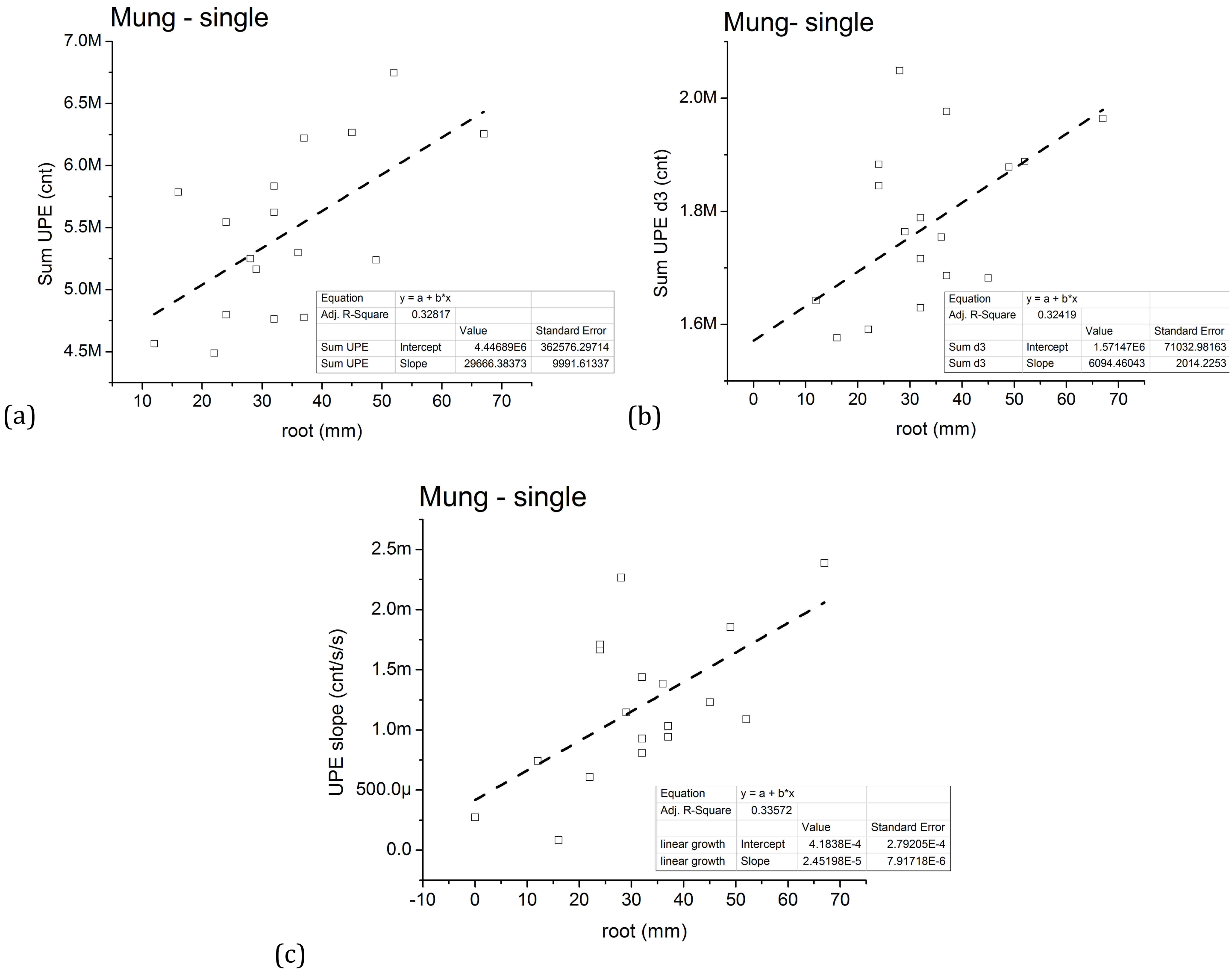

Figure B.1 – Single mung germination tests (m1 to m6) - datagrams of UPE data *versus* the total seedlings' length for the: (a) total photon-count (Sum UPE) for the entire period: 0-72h; (b) counts for 24h period: 48-72h (Sum UPE d3); (c) UPE slope for the total period (0-72h, cnt/s/s); linear approximation with parameters at inset table.

https://doi.org/10.1016/j.jpap.2020.100001

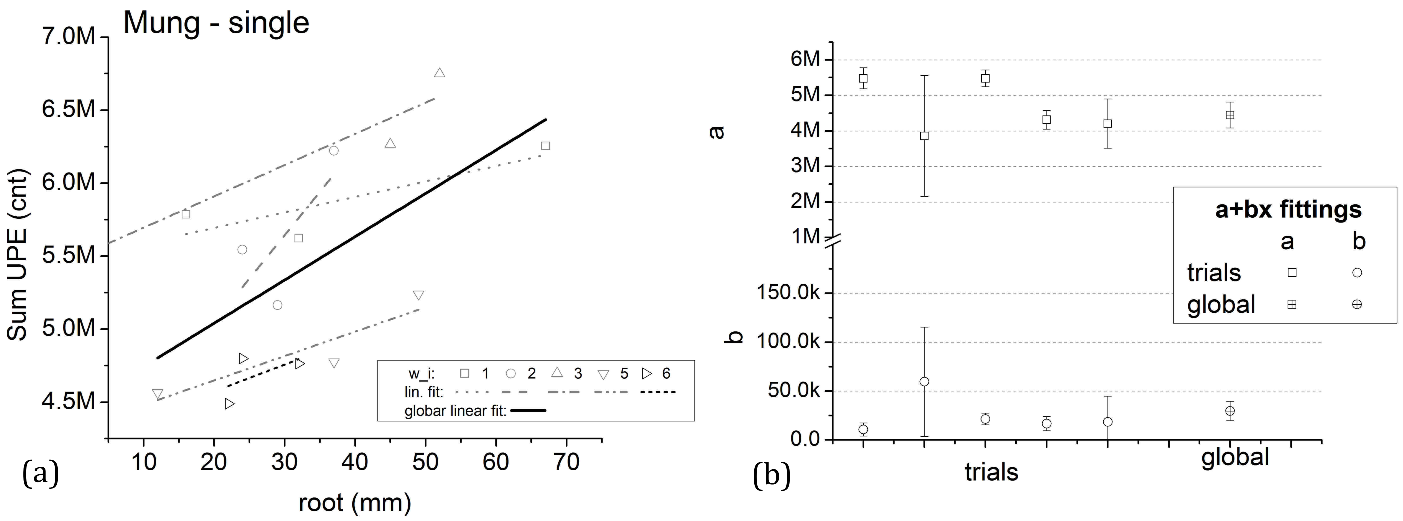

Figure B.2 – Single mung germination tests (excluding m4), total photon-count (Sum UPE) *versus* the total seedlings' length (root): (a) linear fitting for each round and the global one; (b) fitting parameters of (a) plots – the axis intercept 'a' and line slope 'b'.

https://doi.org/10.1016/j.jpap.2020.100001
