# Supplementary material for: Time-resolved ultra-weak photon emission as germination performance indicator in single seedlings
Source: J Photochem Photobiol. 2020 Mar;1:100001. doi: 10.1016/j.jpap.2020.100001 (PMC7446287; doi:10.1016/j.jpap.2020.100001)
Supplement: Supplementary file 3 [file mmc3.pdf]

Appendix C – Single Corn series

Table C.1 – Single corn series

Photon-count time profiles (local average, 1000#), **starting from 72-h** after imbibition (ie. d4 to d6), and photograph at end of the 6-day germination tests of each trial of 3 samples: photon-count chambers ch0, ch1 and ch2 with single corn seedling in petri-dish + 1.5 mL of water (pop corn, Yoki stock G18BRNP23JN). Technical failure led to data lost of the first day of c_5, so it was not used for the datagrams of Fig. S3.1.

Trial	Photon-count profiles	Picture
c_1	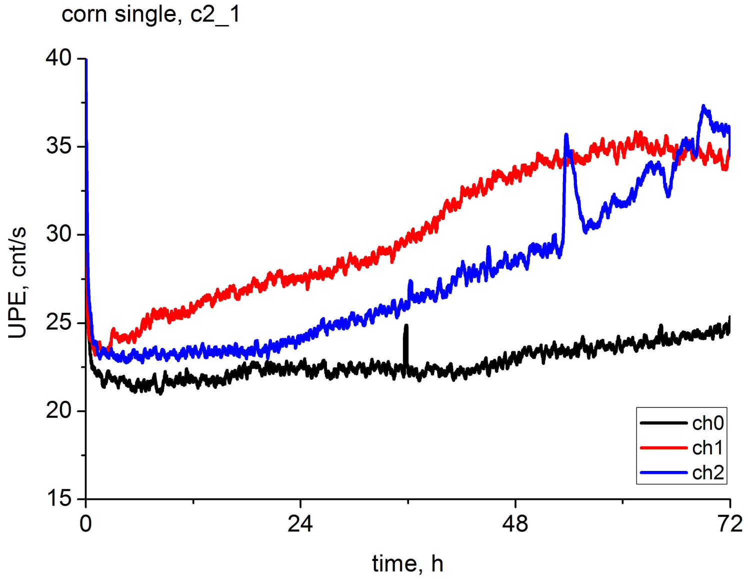	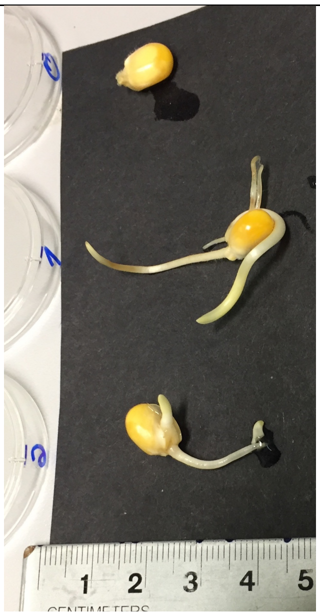
c_2	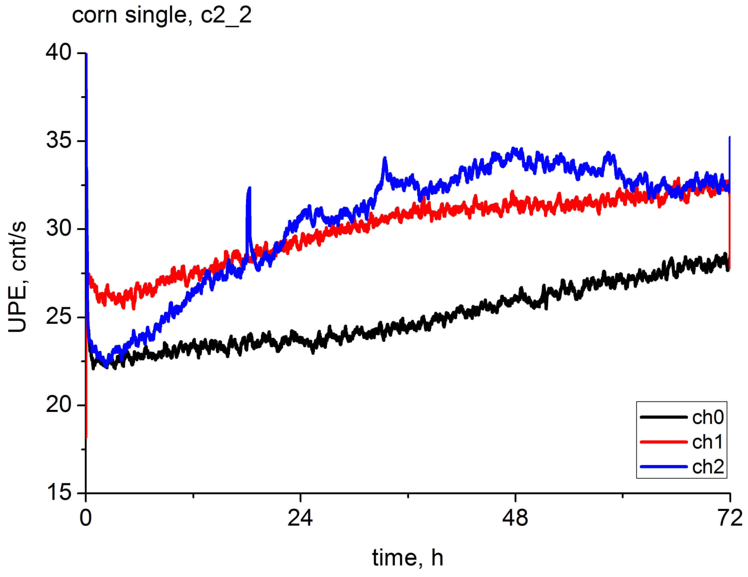	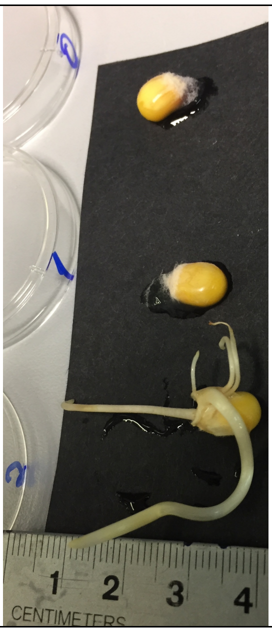

https://doi.org/10.1016/j.jpap.2020.100001

<table>
<tr>
<td>c_3</td>
<td>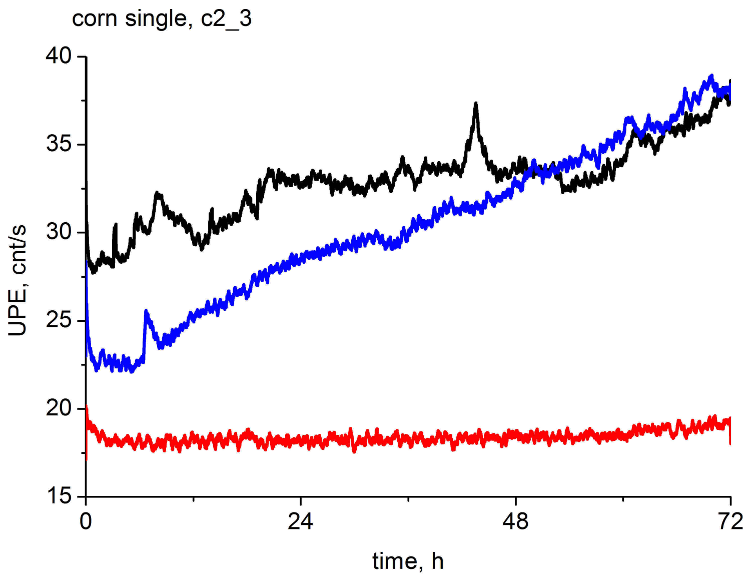
</td>
<td>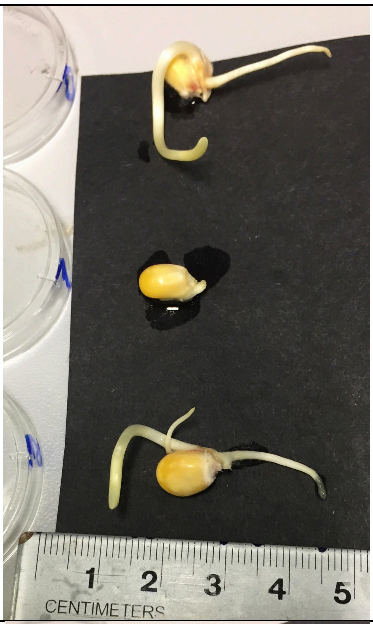
</td>
</tr>
<tr>
<td>c_4</td>
<td>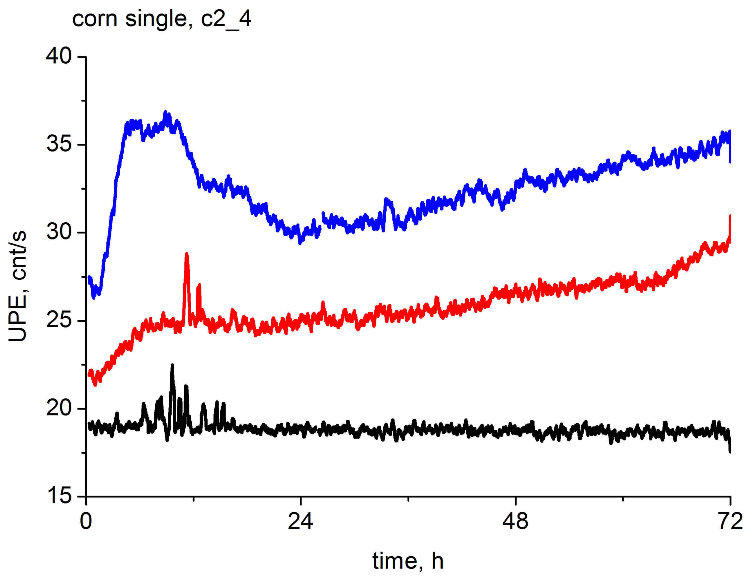
</td>
<td>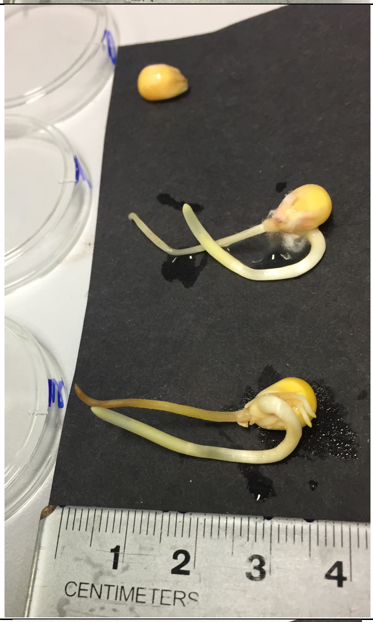
</td>
</tr>
<tr>
<td>c_5</td>
<td>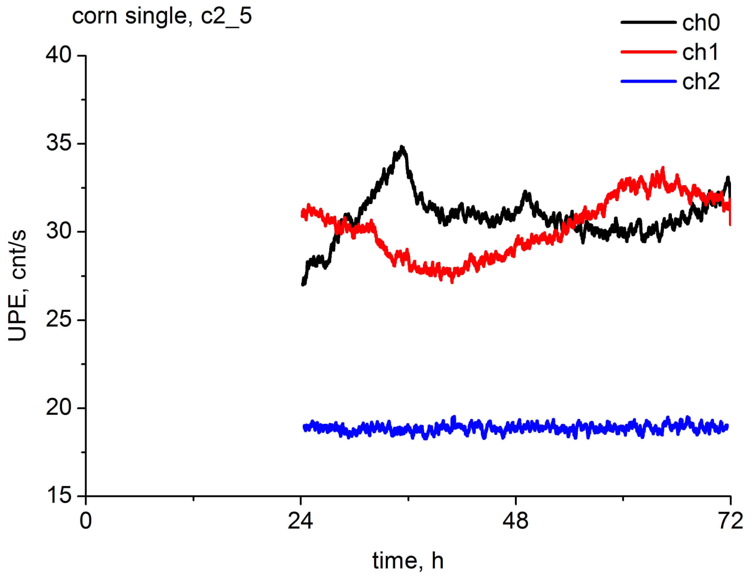
</td>
<td>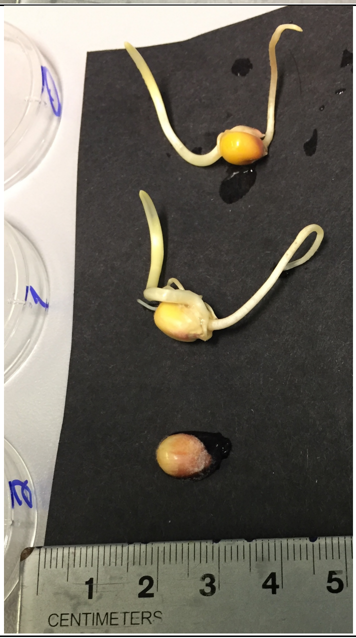
</td>
</tr>
</table>

https://doi.org/10.1016/j.jpap.2020.100001

<table>
<tr><td>c_6</td><td>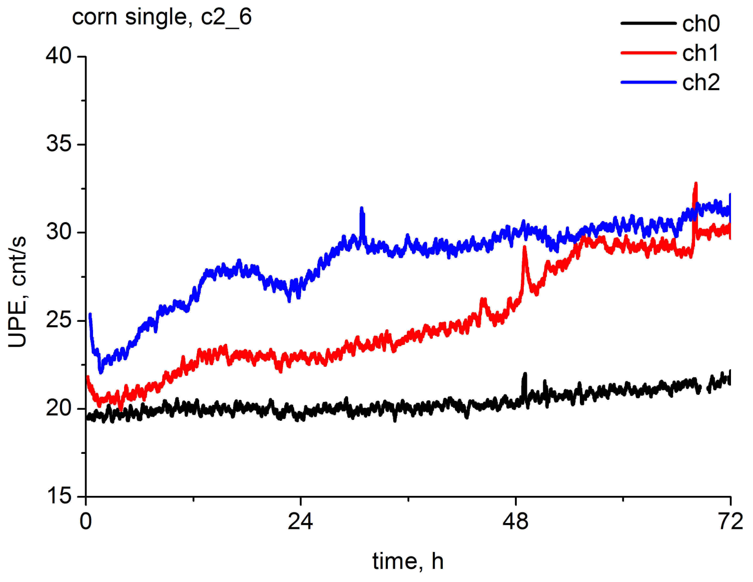
</td><td>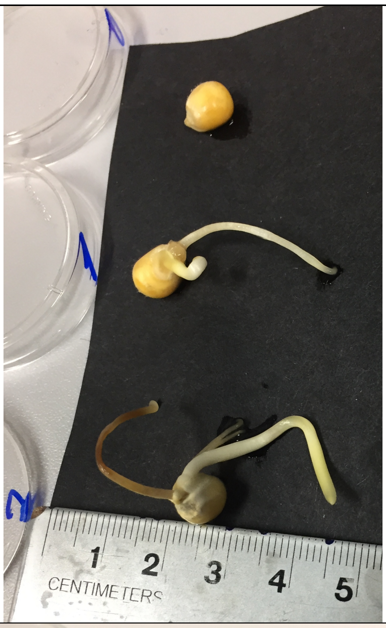
</td></tr>
<tr><td>c_7</td><td>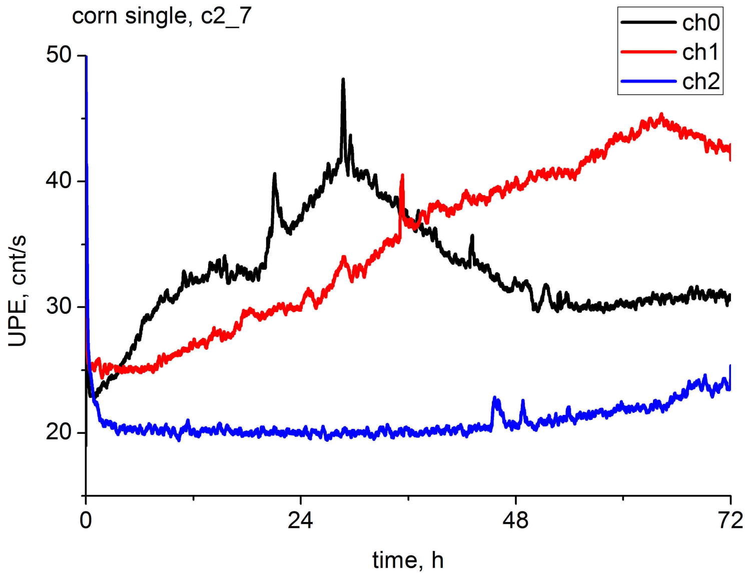
</td><td>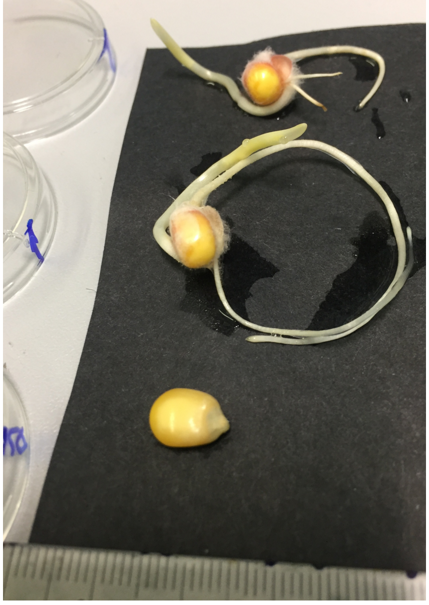</td></tr>
</table>

https://doi.org/10.1016/j.jpap.2020.100001

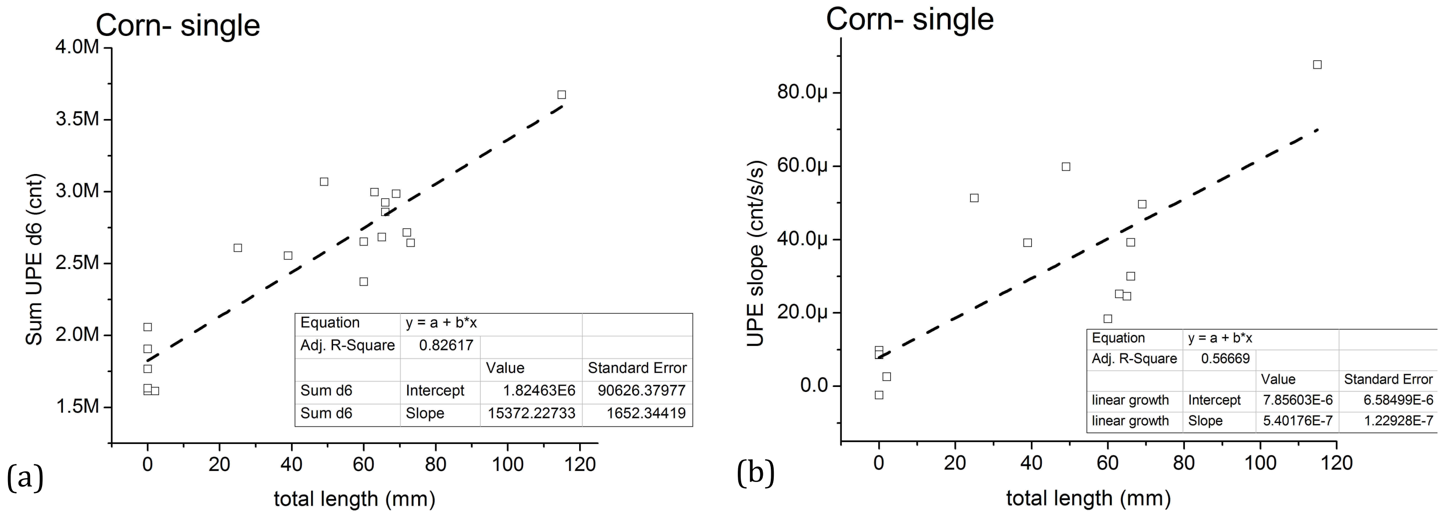

Figure C.1 – Single corn germination tests series (c_1 to c_7) - datagrams of UPE data *versus* the total seedlings' length for the: (a) total photon-count for the last 24h period (Sum d6, 48-72h, cnt); (c) linear growth (Slope, cnt/s/s) of UPE profile for the total period: 0-72h; linear approximation with parameters at inset table.

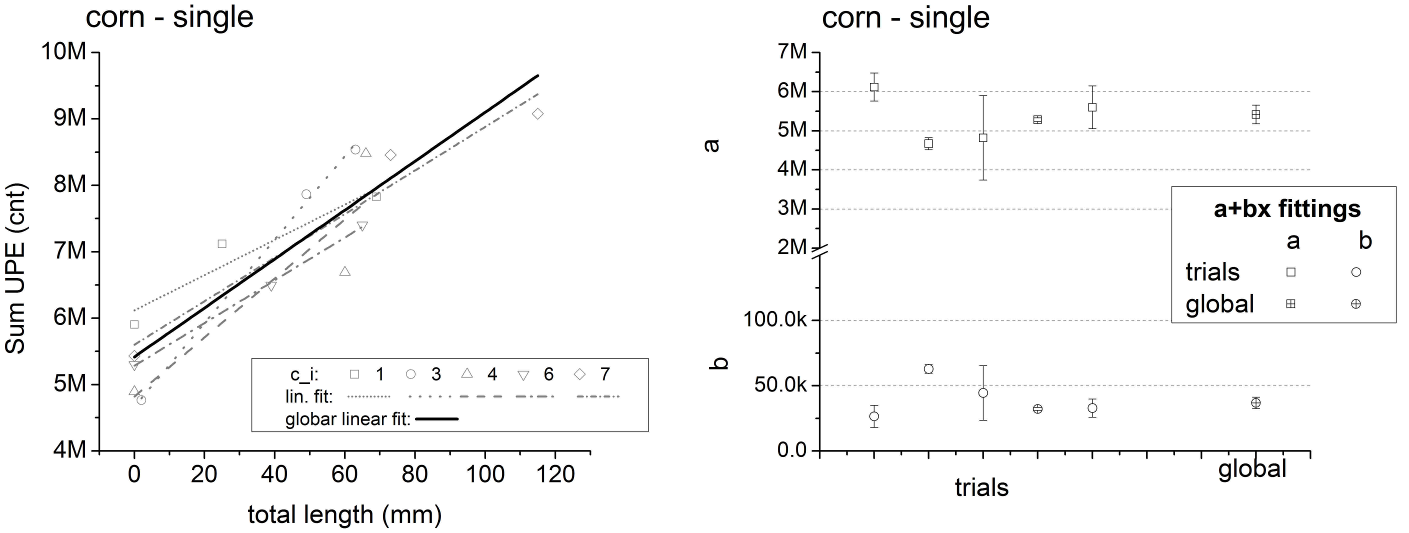

Figure C.2 – Single corn germination tests (excluding c2 and c5), total photon-count (Sum UPE) *versus* the total seedlings' length: (a) linear fitting for each round and the global one; (b) fitting parameters of (a) plots – the axis intercept 'a' and line slope 'b'.

https://doi.org/10.1016/j.jpap.2020.100001
