# Supplementary material for: Time-resolved ultra-weak photon emission as germination performance indicator in single seedlings
Source: J Photochem Photobiol. 2020 Mar;1:100001. doi: 10.1016/j.jpap.2020.100001 (PMC7446287; doi:10.1016/j.jpap.2020.100001)
Supplement: Supplementary file 4 [file mmc4.pdf]

Appendix D– Single wheat series

Table D.1 – Single wheat series w_i

Photon-count time profiles (local average, 1000#) and photograph at end of the 3-day germination tests of each trial of 3 samples: photon-count chambers ch0, ch1 and ch2 with single wheat seedling in petri-dish + 1.5 mL of water (organic wheat grains, *Essential* stock 212036-G125X).

Trial	Photon-count profiles	Picture
**w1**	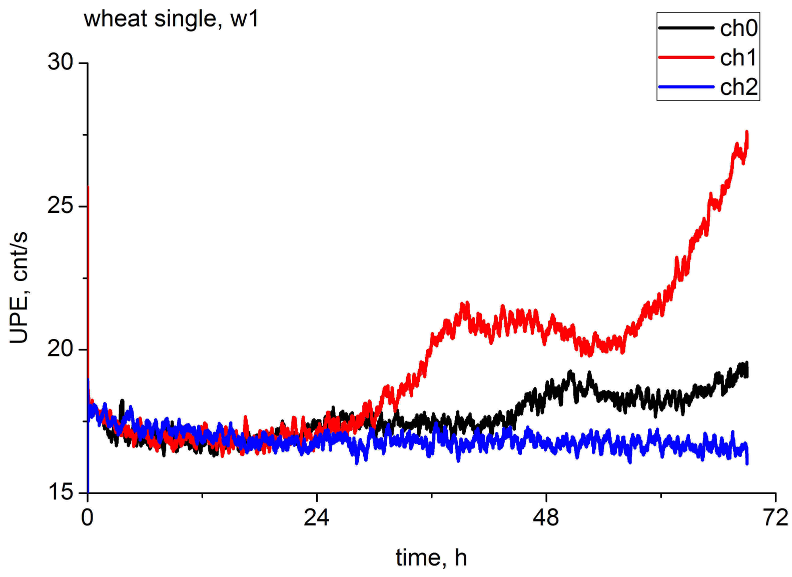	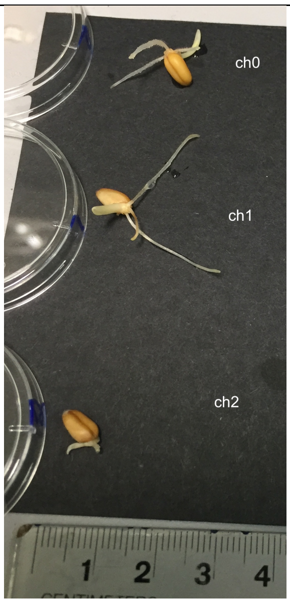
**w2**	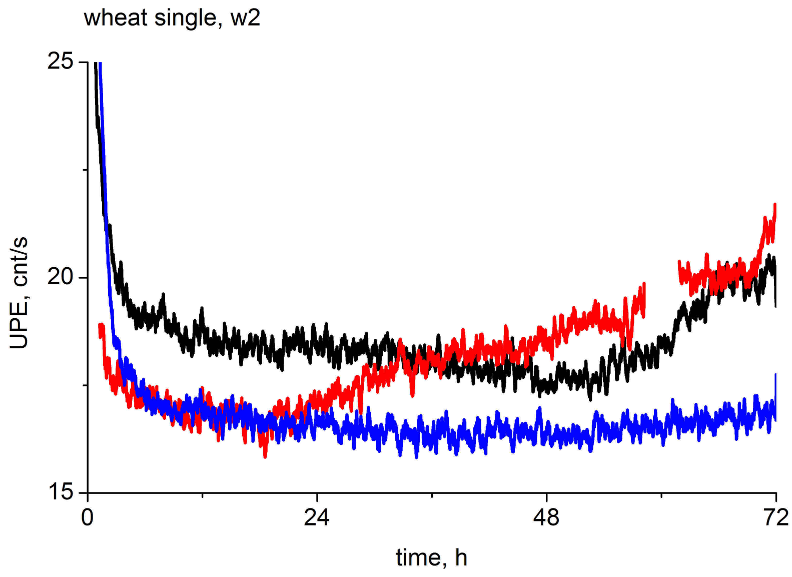	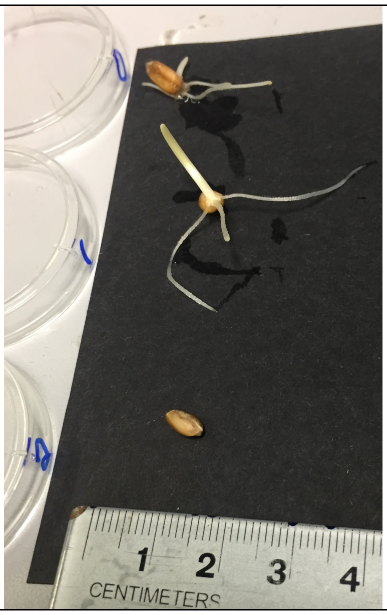

https://doi.org/10.1016/j.jpap.2020.100001

w3

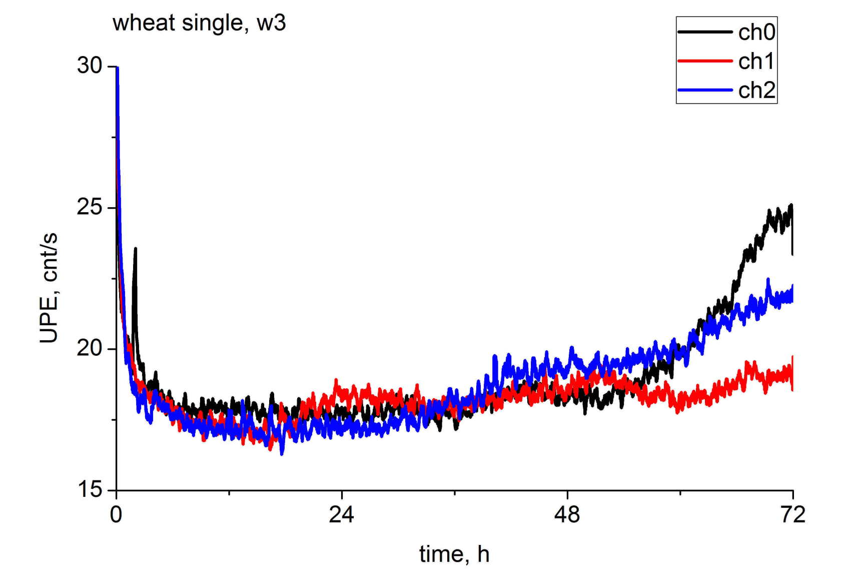

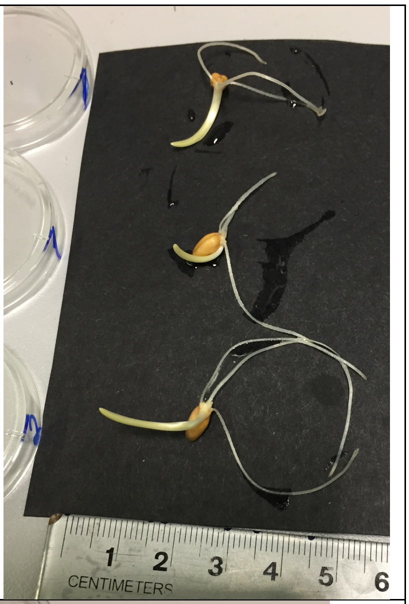

w4

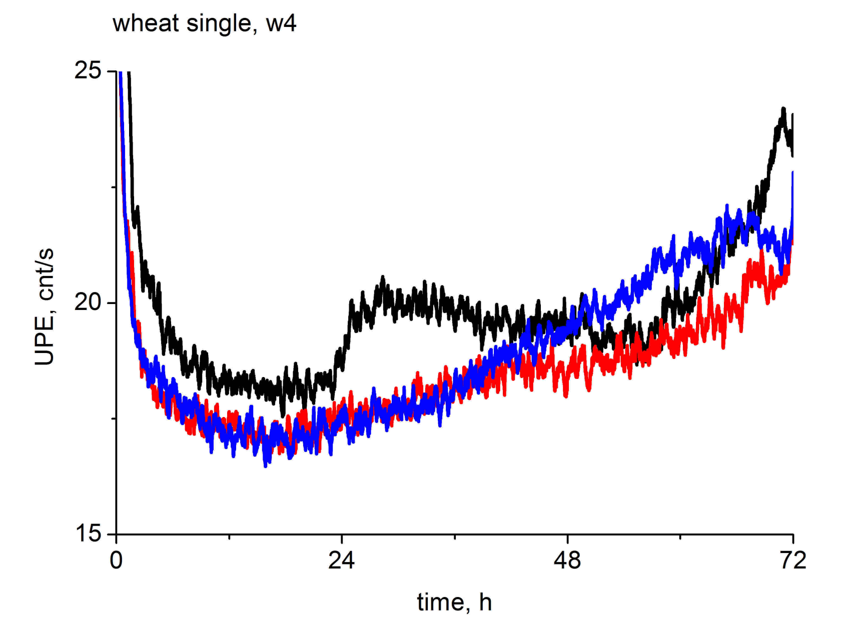

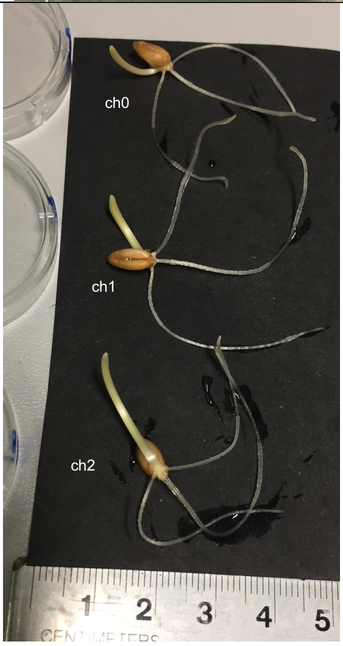

w5

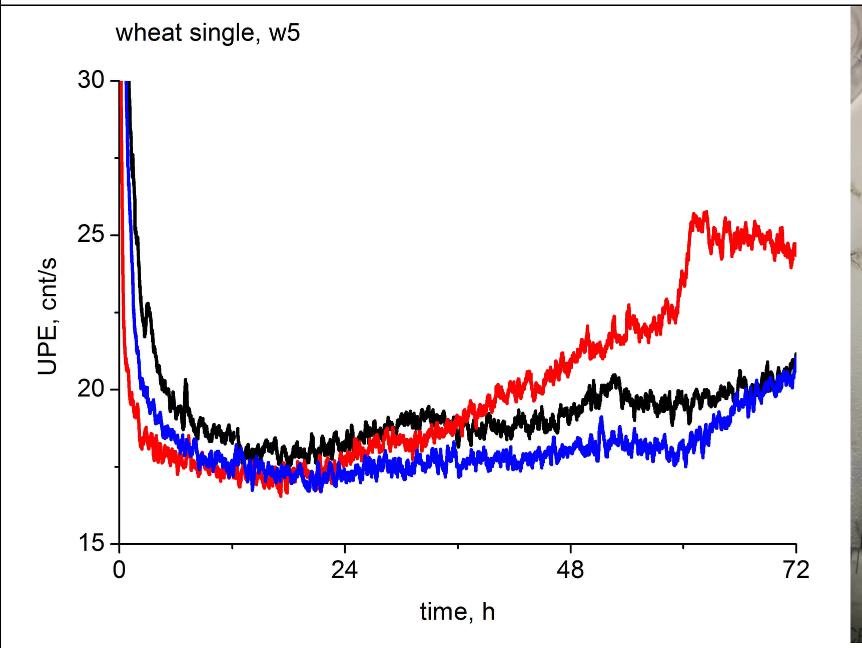

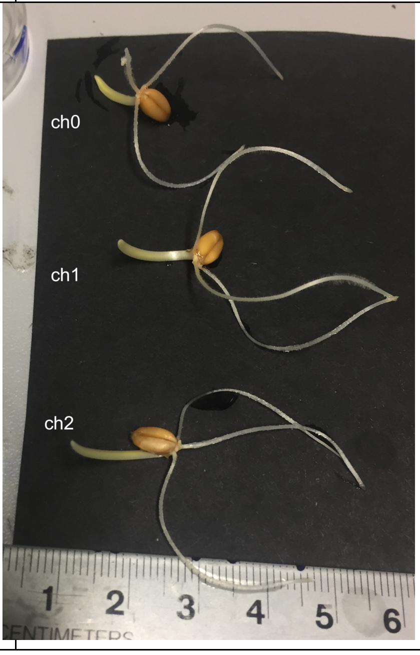

https://doi.org/10.1016/j.jpap.2020.100001

w6

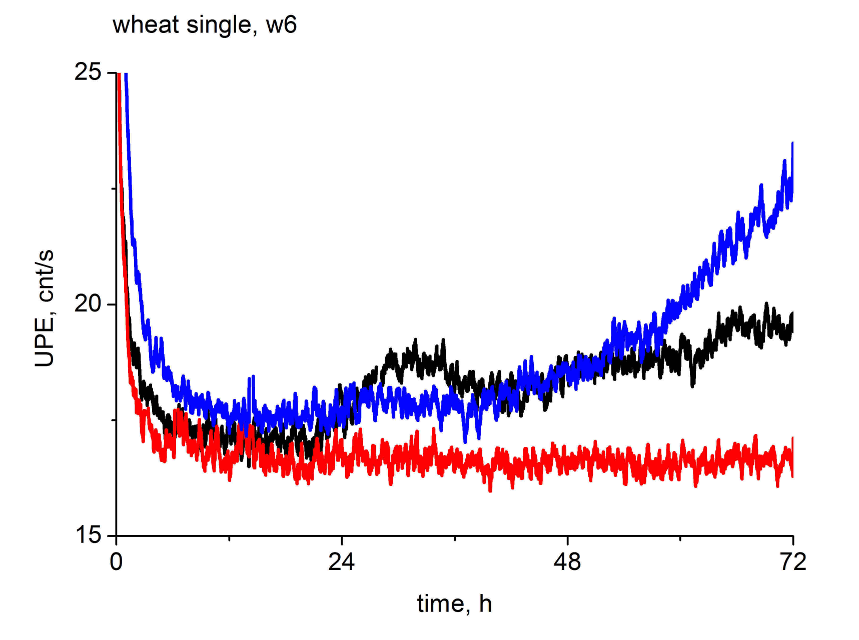

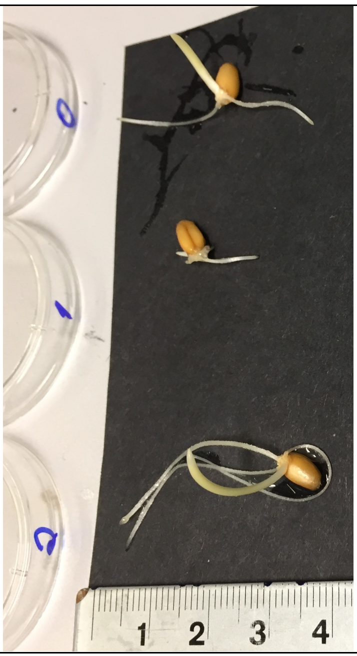

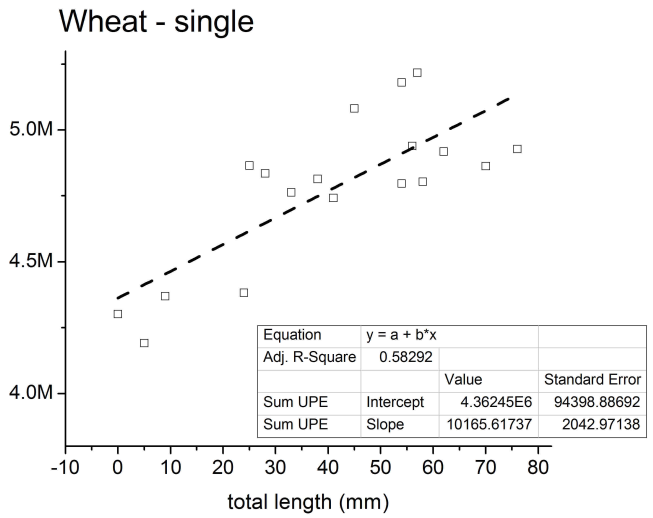

(a)

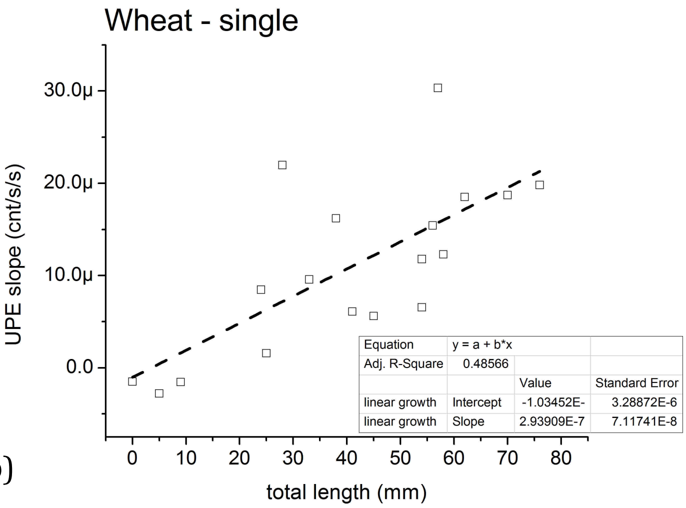

(b)

Figure D.1 – Single wheat germination tests (w1 to w6) - datagrams of UPE data *versus* the total seedlings' length for the: (a) total photon-count (Sum UPE, cnt) for the entire period: 0-72h; (b) linear growth (Slope, cnt/s/s) of UPE profile for the total period: 0-72h; linear approximation with parameters at inset table.

https://doi.org/10.1016/j.jpap.2020.100001

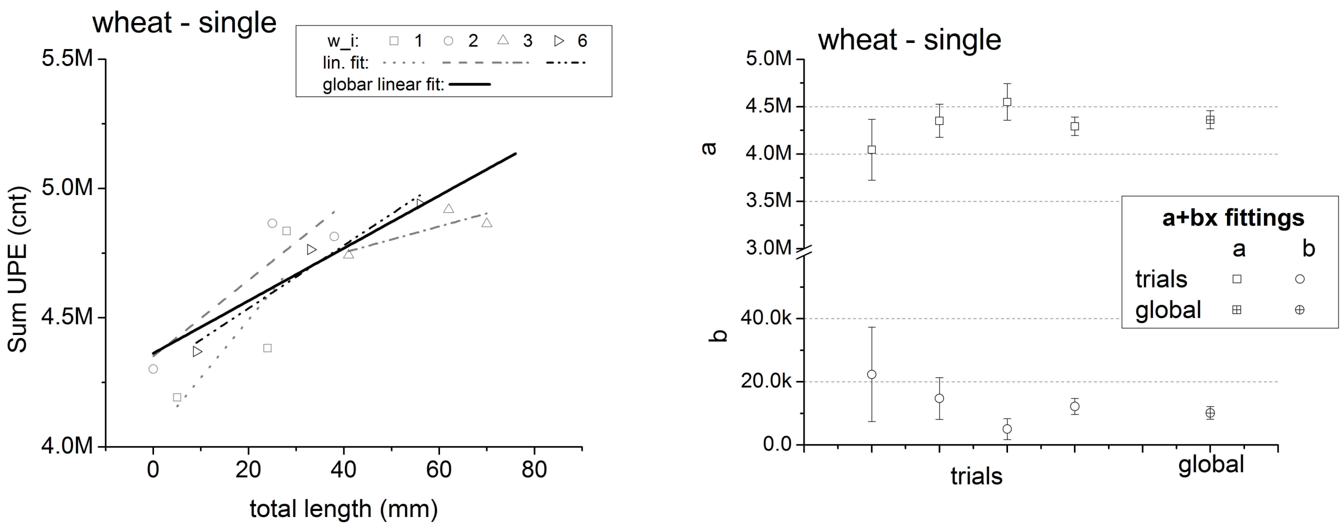

Figure D.2 – Single wheat germination tests (excluding w4 and w5), total photon-count (Sum UPE) *versus* the total seedlings' length: (a) linear fitting for each round and the global one; (b) fitting parameters of (a) plots – the axis intercept 'a' and line slope 'b'.

https://doi.org/10.1016/j.jpap.2020.100001
